# Biological and clinical implications of a model of surveillance immunity

**DOI:** 10.1172/JCI191645

**Published:** 2025-08-01

**Authors:** Katharina Willmann, Luis F. Moita

**Affiliations:** Center for Disease Mechanisms Research, Faculdade de Medicina da Universidade de Lisboa, Lisbon, Portugal.

## Abstract

The immune system must identify genuine threats and avoid reacting to harmless microbes because immune responses, while critical for organismal survival, can cause severe damage and use substantial energy resources. Models for immune response initiation have mostly focused on the direct sensing of microorganisms through pattern recognition receptors. Here, we summarize key features of the leading models of immune response initiation and identify issues they fail to solve individually, including how the immune system distinguishes between pathogens and commensals. We hypothesize and argue that surveillance of disruption to organismal homeostasis and core cellular activities is central to detecting and resolving relevant threats effectively, including infection. We propose that hosts use pattern recognition receptors to identify microorganisms and use sensing of homeostasis disruption to assess the level of threat they pose. We predict that both types of information can be integrated through molecular coincidence detectors (such as inflammasomes or others not yet discovered) and used to determine whether to initiate an immune response, its quality, and its magnitude. This conceptual framework may guide the identification of novel targets and therapeutic strategies to improve the progression and outcome of infection, cancer, autoimmunity, and chronic conditions in which inflammation plays a critical role.

## Introduction

Infection is one of the most serious and frequent threats to an organism that requires defense mechanisms in the form of immune responses. Regardless of their complexity level, effective immune responses share key mechanistic architectures at the sensing, signal transduction, and effector steps. Sensing is arguably the most conserved and important step across organisms, because it determines whether an immune response should be initiated, the most appropriate type, and its magnitude. The decision to initiate or not is critical because immune responses can harm the host, potentially causing substantial tissue damage or autoimmunity, resulting in organ failure or death (1). Moreover, immune responses are energetically demanding and consume limited resources (2). Therefore, sensing bona fide pathogens while avoiding responses against harmless microorganisms is essential to minimize negative effects on fitness and promote host survival (3). The standard immune response initiation models have mostly focused on directly sensing microorganisms through their molecular motifs via pattern recognition receptors (PRRs). While PRRs have been central to understanding the pathophysiology of immune responses, they sense molecular signatures shared between pathogens and commensal organisms and therefore cannot serve to distinguish them.

**Figure 1:**
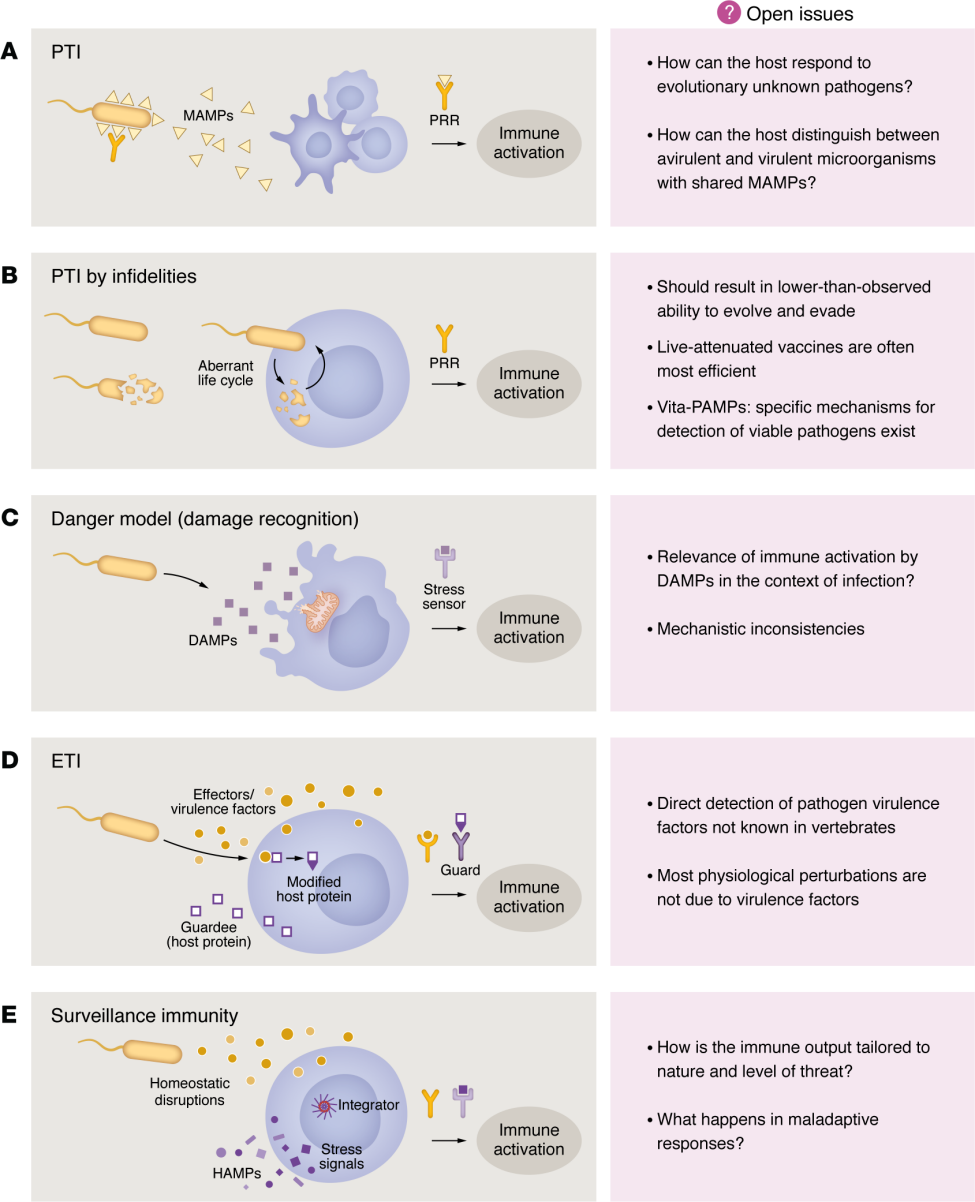
Models of innate immune initiation. (**A**) Pattern-triggered immunity (PTI). Microbial structural molecules (PAMPs or MAMPs) are directly sensed by PRRs, which can activate transcriptional programs or effectors directly. MAMPs that are not conserved or are unknown to the host may not activate PTI. MAMPs may be shared between virulent and avirulent microorganisms (102). (**B**) PTI by infidelities (14). This model proposes that PRRs are predominantly byproducts of unsuccessful pathogens that lead to biochemical infidelities. This implies a high pressure on pathogens to minimize unsuccessful events and should result in a lower-than-observed ability to evolve and evade (14). Additionally, live-attenuated vaccines tend to have the highest efficiency and sensing of markers of live pathogens (vita-PAMPs) by the host (103). (**C**) Danger model (damage recognition) (16). PRRs are activated by sensing host molecular patterns released upon compromised tissues. The relevance of DAMPs in the context of infection has not been fully resolved in this model. (**D**) Effector-triggered immunity (ETI) (21). Virulence factors are sensed by “guard proteins” directly or indirectly by detecting changes or modifications in host proteins (“guardees”). (**E**) Surveillance immunity (3). Immune responses are triggered by disruption of core cellular functions or homeostasis parameters through stress pathways. Multiple input pathways synergize to generate an output tailored to the nature and level of threat. However, maladaptive responses cannot be fully avoided. Yellow symbols depict microbial factors; purple symbols depict host factors. HAMPs, homeostasis altering molecular processes.

Unlike commensals, pathogens disrupt homeostasis in their hosts, often expressed by signs and symptoms of infection. Substantial changes in the set points of strictly controlled physiological parameters are an inevitable consequence of the pathogen’s invasion and life cycle. The information resulting from sensing these changes is likely to integrate with signals that directly identify the presence of a microorganism to shape the decision to initiate an immune response, its quality, and its magnitude. This Review presents a conceptual framework of surveillance immunity that integrates a critical role for mechanisms that sense homeostasis deviations resulting from infection with the initiation and regulation of immune responses. Before we explore this model and discuss its biological and clinical implications, we will briefly describe key features of theoretical models of immune response initiation and identify their main insufficiencies (Figure 1).

## Models of immune response initiation

Theoretical models are critical to organizing observations into coherent, explanatory, and predictive frameworks that generate new research hypotheses. Development of these models represents a critical step for new biological discoveries and the potential identification of novel therapeutic approaches informed by knowledge generated from their use.

### Pattern-triggered immunity model.

The pattern-triggered immunity (PTI) model has been the standard framework for over three decades to explain the mechanisms of immune response initiation (4). From the early formulation of PTI, it follows that germline-encoded PRRs recognize evolutionarily conserved microorganism-associated molecular patterns (MAMPs) (5), motifs associated with molecules that are essential for microorganisms’ survival, but are not produced by the host organism (6). Classic examples are LPS and flagellin, but MAMPs can be functionally and structurally very diverse, ranging from several types of nucleic acids with microbe-specific modifications to proteins, lipids, and carbohydrates (7). PRRs are grouped into families mostly according to their targets and include TLRs, NOD-like receptors (NLRs), RIGI-like receptors (RLRs), other cytosolic nucleic acid sensors, and C-type lectin receptors (CLRs) (reviewed in ref. 7). PRR activation triggers immune signaling pathways that initiate gene expression and metabolic programs, leading to effector responses tailored against each pathogen group, ultimately leading to the generation of long-lasting adaptive immunity (6). These programs are under the control of critical pathways, such as NF-κB, MAPKs, and interferon-regulatory factors (8), that transcribe immune effector molecules like chemokines, cytokines, and interferons and orchestrate an immune and inflammatory response. Other PRRs, like the NLRs, assemble in multimeric complexes, such as the NLR-mediated inflammasome that activates caspases and converts molecular precursors into their bioactive forms.

Experimental observations extensively support this PTI model’s key conceptual components. It has had foundational importance in the field of innate immunity, guiding the mechanistic dissection of its core principles. However, PTI has not resolved central problems in immune response initiation, particularly how the host distinguishes commensals from pathogenic microorganisms. MAMPs are shared by all microorganisms within a specific group, not restricted to pathogens (9, 10). In addition, MAMPs are often described as evolutionarily conserved molecules that microorganisms cannot molecularly change because even small modifications would compromise their viability. However, extensive variations of MAMPs within each group, or even species, are frequent and represent a strategy for pathogens to avoid or antagonize detection (9, 11). The recent demonstration that organisms fail to respond to pathogens with known MAMPs but no common evolutionary history further supports this argument (12). The barcode hypothesis proposes that microorganisms of high pathogenic potential could be recognized by their distinct combinations of MAMP patterns, which would allow for tailor-made responses (13). However, such combinations cannot categorize most pathogens, suggesting additional cues for pathogen distinction are necessary (9).

Therefore, the PTI model is insufficient to explain how hosts distinguish between infection and colonization, viable and dead, and pathogenic and nonpathogenic microorganisms. It also does not provide mechanistic insights into how the host assigns the quality, magnitude, and duration of an immune response in the face of a perceived infectious threat. Therefore, PRR activation is not sufficient. Additional signals are required to shape an effective immune response and to minimize collateral tissue damage.

### Infidelities model.

According to the infidelities model, PRRs detect pathogens via MAMPs, often resulting from microbial biochemical *infidelities* or mistakes during the infectious life cycle (14). Incomplete or erroneous microbial processes, like the release of incomplete viral genomes or misdirected bacterial components, still activate immune responses. This model proposes that PRRs may not target pathogens directly but instead detect the products of such errors (14). Although decreasing these errors would minimize detection, some level of error is critical for microbial evolvability (and, consequently, survival), so pathogens cannot eliminate or even excessively lower their rate (14). The model posits that exceptions, including dedicated TLR detection of functional LPS, are relatively recent evolutionary events. If correct, the infidelities model’s predictions may guide the development of more effective immunotherapies and antibiotic drugs. Novel strategies could be based on superior modes of PRR activation or on targeting pathogen infidelities. However, this model is not supported by the observation that live-attenuated vaccines are more effective at generating protective immune responses than those that use inactivated or subcomponents of the pathogen. These observations instead support the idea that sensing indicators of pathogen viability and infectivity synergize with MAMP sensing to elicit effective and vigorous immune responses (discussed below).

### Patterns of pathogenesis.

**Figure 2:**
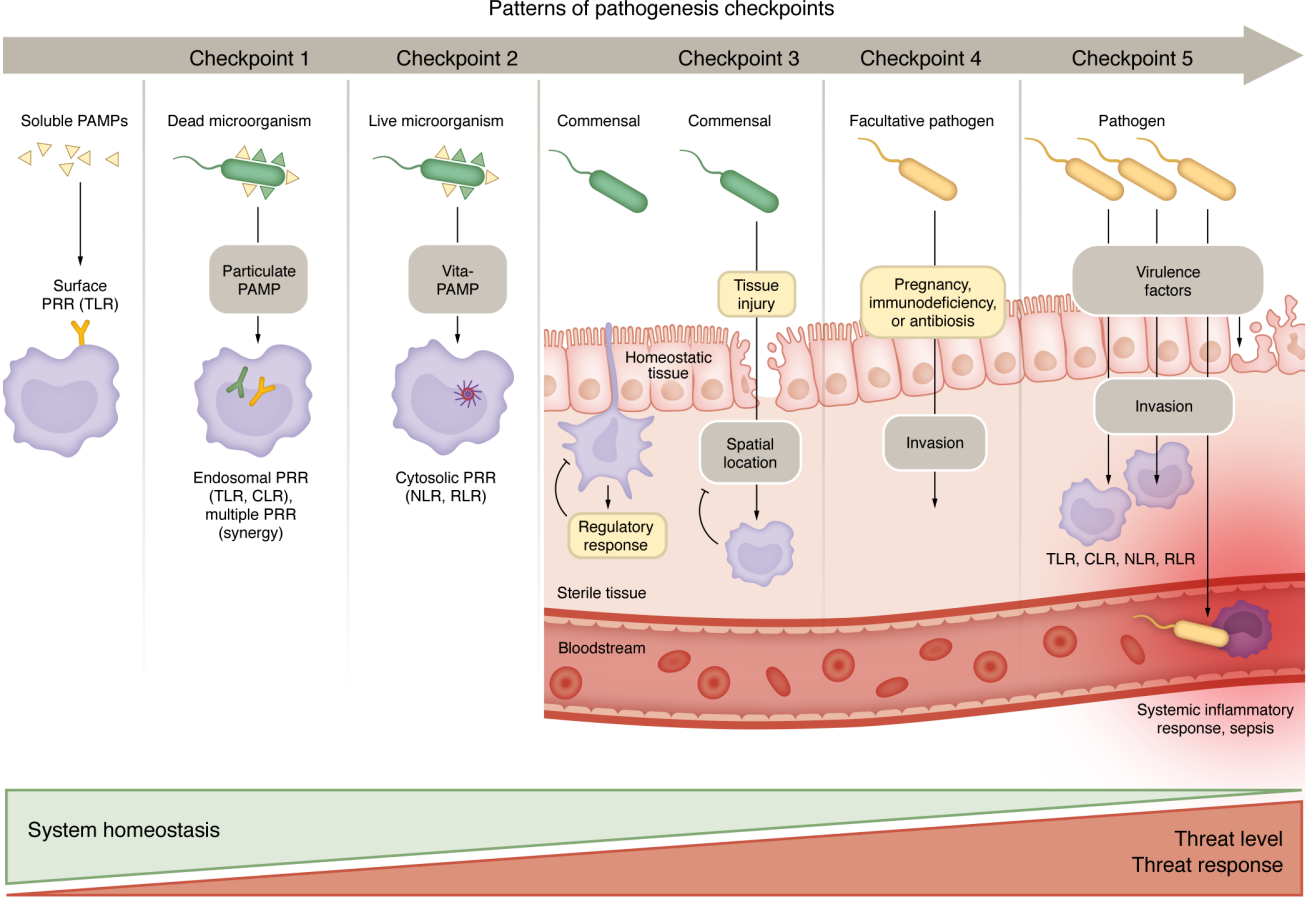
Patterns of pathogenesis. The risk level (threat to host system homeostasis) and the magnitude and nature of the immune response that needs to be activated are assessed using direct sensing of microorganisms and additional contextual signals (9). The pathogen must overcome several checkpoints (depicted in columns labeled Checkpoint 1–5) before it poses the highest level of threat, resulting in a vigorous immune response (7). Checkpoint 1: Soluble MAMPs initiate cytokine and chemokine production remotely, while MAMPs on whole microorganisms trigger direct microbicidal responses. Checkpoint 2: Vita-PAMPs, such as bacterial mRNA, indicate live microorganisms capable of growth, multiplication, and invasion and trigger enhanced immune responses by activating PRRs. Checkpoint 3: The need and type of immune response to microbial presence varies according to the tissue’s physiology and microenvironment, ensuring appropriate responses. Systemic threats trigger immediate, strong reactions to prevent severe consequences, while local tissue responses are tightly regulated. At the subcellular level, the strongest immune responses are initiated against agents that invade the cytosol. Checkpoint 4: The degree of invasiveness is critical information for the immune system to distinguish between pathogenic and nonpathogenic microorganisms. While commensal bacteria coexist with the host without causing disease, they can become pathogenic if they breach sterile tissues. Invasive forms of microbes expose specific molecules or morphologies that signal potential threats, leading to more robust immune activation. Commensals can act as facultative pathogens under specific conditions. Commensal bacteria can become invasive due to host factors like immunodeficiency, pregnancy, or treatments altering the microenvironment. The immune system and intact physical barriers are crucial for preventing this switch. Invasiveness can be controlled by inhibiting the quorum-sensing system of microorganisms. Checkpoint 5: Virulence. Microorganisms are classified as pathogens or nonpathogens based on their ability to cause disease, correlating with virulence factors that disrupt host barriers and invade tissues.

The patterns of pathogenesis (9) concept proposes that the immune system responds to MAMPs by contextualizing additional signals. Directly sensing microorganisms alone is insufficient for distinguishing pathogens from commensal microorganisms and selecting an appropriate immune response. Additional signals may derive from factors that pathogens use to infect their hosts, multiply, and later spread to additional hosts. Sensing additional microorganism characteristics and the consequences of their presence may help hosts form an assessment of pathogen virulence (Figure 2), influencing immune response initiation and calibration based on the threat level. For example, virulence factors like pore-forming toxins and bacterial secretion systems may strongly signal pathogenicity and activate an immune response (reviewed in ref. 15). It has been proposed that the threat level to the host and the necessary immune response quality and magnitude can be assessed by integrating at least five checkpoints (Figure 2, reviewed in ref. 7). These include the integration of tissue-specific signals and the distinction between (a) soluble and particulate MAMPs, (b) viable and dead microorganisms, (c) appropriate spatial location of microorganisms, (d) invasive and noninvasive microorganisms, and (e) pathogenic and nonpathogenic microorganisms. Within the framework of patterns of pathogenesis, the danger, the effector-triggered immunity, and the surveillance immunity models describe possible paradigms of immune response initiation.

### Danger model.

The danger model (16) states that the immune system recognizes pathogens through the consequences of their presence by identifying damage-associated molecular patterns (DAMPs), endogenous molecules released from host cells due to cell death or damage (reviewed in ref. 17). The immune system would recognize the damage caused by pathogens, not the microorganisms that cause it (18). This model has been useful in the context of sterile inflammation but is insufficient in infection, where mechanistic inconsistencies, especially its initiation step, remain unresolved (18). It will be interesting to investigate mechanisms through which the host may be able to distinguish between sterile injury and injury caused by pathogens or the immune response to eliminate them.

### Effector-triggered immunity model.

The effector-triggered immunity (ETI) model was initially defined in plants as a protective type of immune response against microbial effectors (19). Because PRR signaling alone provides insufficient information about microorganismal threat level, sensing virulence factors is a critical component of immune response initiation (Figure 2) (15, 20). Detection of pathogen-encoded virulence factors most often occurs indirectly through the sensing of their virulence activities (reviewed in ref. 21). Examples are (a) the guard hypothesis (22), wherein a virulence factor can modify a target protein (the “guardee”) that is identified by a sensor (the “guard”); (b) a virulence factor is directly identified by a host sensor (an example mostly restricted to the case of plants); (c) a virulence factor causes cellular stress; and (d) the pathogen activity perturbs or eliminates a protein that is an inhibitor of immune responses. ETI was reviewed in ref. 21 and, therefore, will not be analyzed in detail here. Although the scope of ETI is expanding (15, 20), creating considerable conceptual overlap between ETI and surveillance immunity, here we take the stricter definition of ETI, proposing that virulence factors from pathogens are the key features used for their recognition (15, 20), distinguished from surveillance immunity pathways initiated by a broader range of stimuli, even those beyond infection (discussed below).

**Figure 3:**
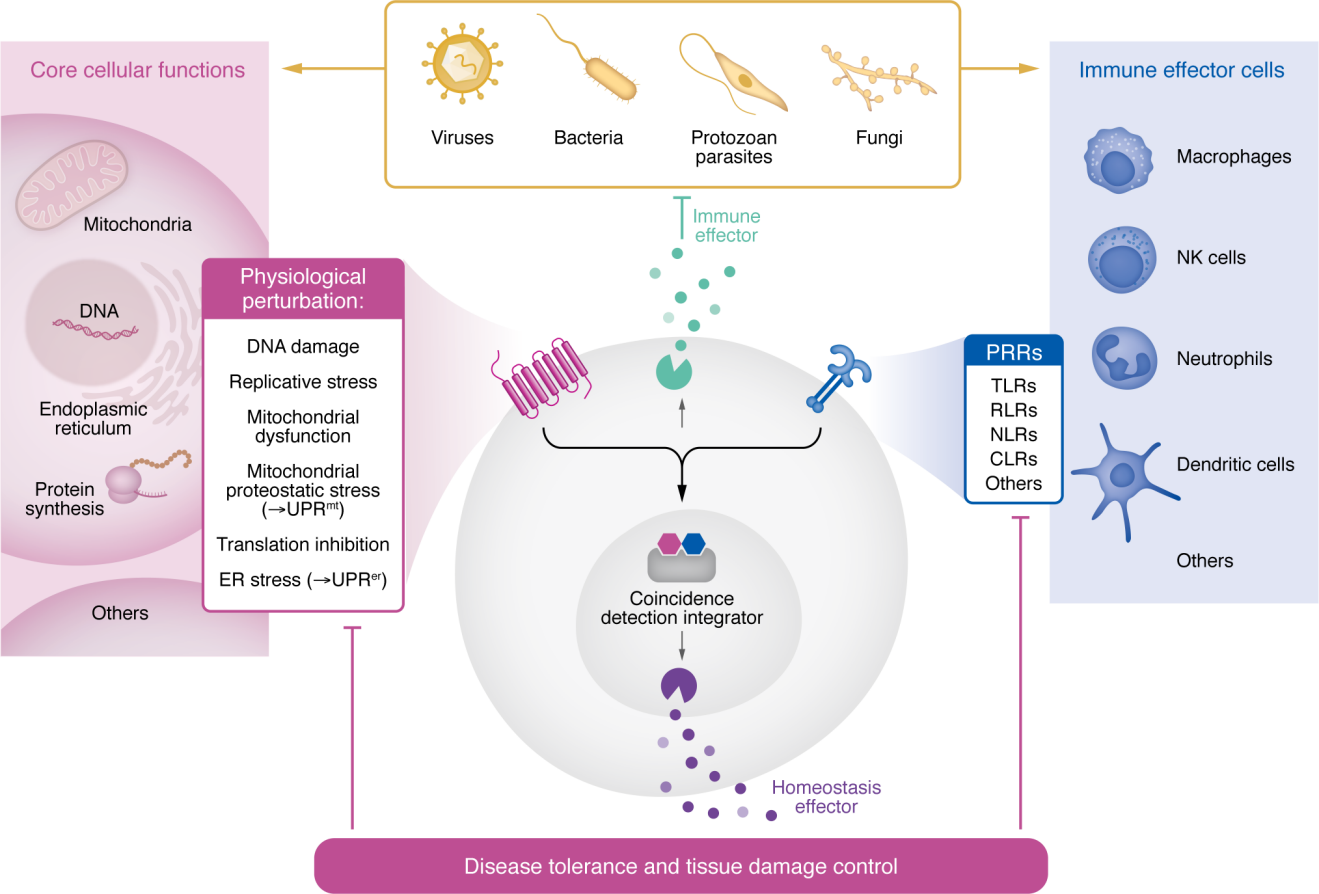
Surveillance immunity. All major groups of pathogens (viruses, bacteria, protozoan parasites, and fungi) trigger stress responses to core homeostatic processes such as DNA damage, replicative stress, mitochondrial dysfunction and proteostatic stress (UPR^mt^), translation inhibition and ER proteostatic stress (UPR^er^), in addition to direct recognition by PRRs. Direct and indirect sensing of homeostasis disruption and signaling by PRR is integrated and synergizes in the production of immune and homeostasis effectors to tailor effector responses to specific classes of pathogens and level of threat. Both disease resistance (directed against the pathogen) and disease tolerance mechanisms that act on the host (to limit tissue damage, collateral damage, and tissue dysfunction) are activated. ER, endoplasmic reticulum.

Overall, the models summarized above do not resolve key questions relating to immune response initiation. In recent years, several other theoretical models that integrate physiology principles have been proposed to address these issues and attempt a unifying and coherent framework where immunity is a central component of the many physiological processes that maintain homeostasis. These include a framework for homeostasis maintenance (23), the discontinuity theory of immunity (24, 25), the equilibrium model (26–28) and the quantal theory of immunity (29). We (3) and others (30, 31) have previously proposed a model of surveillance immunity for the initiation of innate immune responses and homeostasis maintenance. In the following sections, we describe, update, and discuss the biological and clinical implications of this model (Figure 3) that focuses on the central role of physiological disruptions for the initiation and quality of immune responses.

## Surveillance immunity model hypothesis

Homeostasis is a dynamic and self-regulating process that allows organisms to actively maintain a stable internal environment in response to changing internal and external conditions, using negative feedback mechanisms (3, 32). Survival and functional preservation require keeping several physiological parameters within strictly enforced ranges (3, 32). First-line homeostatic circuits are disrupted in response to large internal or external perturbations, such as severe systemic infection. In these cases, negative feedback mechanisms are insufficient to maintain key regulated variables within the required ranges (33) and need instead feed-forward mechanisms like inflammation that coordinate emergency responses to restore homeostasis (34) but may activate a hyperinflammatory state in the host as a result.

### Surveillance immunity model.

Our proposed surveillance immunity model hypothesizes that organisms integrate the information coming from the direct sensing of a microorganism, mostly via its molecular signatures using PRRs, with information about physiologic disruption resulting from the activities of the pathogen. This enables the host to assess the level of threat and gauge the need to initiate an immune response, its quality, and magnitude. We predict that while initial physiological perturbations will be caused by the pathogen alone, once the immune response is initiated, immune-driven physiological disruptions (including those caused by collateral tissue damage or cytokine production) will also contribute to a possible feed-forward process to shape and amplify the immune response or to terminate it. For example, some cytokines will induce fever, which is known to promote pathogen control by many processes, including increasing T cell proliferation, cell migration, and antigen presentation and restricting pathogen replication (35).

In the extreme, organisms may initiate inflammation and full-blown immune responses owing to substantial deviations in homeostasis alone (30). This framework implies that initiating an immune response requires contextual information that accompanies the presence of a microorganism. The contribution of additional signals, or even their self-sufficiency for immune response initiation, is particularly well illustrated by the fact that organisms that lack bona fide PRRs, like *Caenorhabditis elegans*, initiate aversive behaviors to pathogens and are capable of mounting effective immune and detoxification responses against them (30). Multiple pathways for detoxification, pathogen response, and mitochondrial repair were first discovered in *C*. *elegans*, including ceramide biosynthesis and the mevalonate pathways (36), and nuclear hormone receptor-dependent detoxification genes (37). Interestingly, mitochondrial dysfunction triggers RNA interference in *C*. *elegans* through a pathway homologous to the mammalian RIG-I antiviral response (38).

The initiation of immune responses based on the information provided by sensors of substantial physiological disruption may be able to detect a wide range of relevant threats, regardless of the initiating molecular signatures, using a small set of genome-encoded components (3). In addition to providing a critical contribution to the initiation step of an immune response, it is likely that early sensing of substantial deviations in homeostasis parameters can also play a critical role in limiting tissue damage and the activation of tissue damage repair to preserve organ function and to allow the return to steady state (39).

We propose that coincidence detectors may mechanistically mediate the integration of signals from PRRs and sensors of homeostasis disruption (Figure 3). The inflammasome is currently the best example to function as a coincidence detector. The effector function of inflammasomes requires two signals. The first leads to NF-κB activation and may be initiated by directly sensing microorganisms using PRRs. The second activating signal is often given by potassium efflux across the plasma membrane resulting from the effect of microbial toxins that disrupt ion gradients across the membrane, as in the case of *Staphylococcus aureus* α-toxin (40), which can indicate membrane disruption by pathogen activity. This type of mechanism applies not only to the plasma membrane, but also to intracellular membranes; for instance, the influenza virus M2 protein is a proton-selective ion channel that neutralizes the pH of the trans-Golgi network (41). Other good candidate molecules for coincidence detectors, including those with a known role in immune responses, are the TIR-domain-containing adaptor protein-inducing IFN-β (TRIF), as well as the PRRs NOD1/2, which sense bacterial peptidoglycans (42). In addition to their role as PRRs, NOD1/2 also act as metabolic sensors of stress by responding to the endogenous metabolite sphingosine-1-phosphate (S1P) that is produced in response to cellular perturbations (43). Notably, while S1P binds directly to the nucleotide-binding domains of NOD1/2 to activate NF-κB signaling, peptidoglycan sensing is achieved through the leucine-rich repeats domain of NODs (43), meaning that a PRR molecule can act as a coincidence detector integrating bacterial and metabolic cues for optimal activation of downstream inflammatory responses. TRIF can also act as a coincidence detector by linking the TLR and NLRP3 inflammasome pathways using a different mechanism: TRIF is critical for production of high concentrations of IFN-β in response to the mRNA vita-PAMP, which distinguishes dead from live bacteria and informs the host organism on the threat posed by the presence of a microorganism (44). One of the main downstream effector roles of coincidence detectors like TRIF and inflammasomes may be the coupling of transcriptional and posttranslational processes, possibly initiated by different signals resulting from sensing of MAMPs and physiologic disruption, which are required to produce most immune effectors.

Another key prediction of the surveillance immunity model is that pathogens, unlike commensals, cause substantial metabolic stress in the host. Tightly regulated self-metabolites, which can be sensed directly or indirectly, undergo large deviations from the homeostatic set points. For instance, as described above, NOD1/2 sensing of S1P results from cellular stress that increases in response to disruption of cellular homeostasis by the presence of a pathogen (43). In addition to sensing self-metabolites, the host can directly sense a repertoire of non-self-metabolites produced by microorganisms, which may serve as a measure of their pathogenic potential and, therefore, distinguish commensals from pathogenic microorganisms. For example, *C*. *elegans* uses the NHR-86 nuclear hormone receptor (a homolog of mammalian hepatocyte nuclear factor 4 [HNF4], which has roles in glucose and lipid metabolism in insects, ref. 45) to sense the non-self, toxic pathogen–derived phenazine-1-carboxamide metabolite produced by pathogenic strains of *Pseudomonas aeruginosa* and activate innate immune responses (46). In addition, *C*. *elegans* can initiate a behavioral avoidance response of pathogens following the olfactory sensing of volatile compounds from pathogens like *P*. *aeruginosa* (47). An avoidance response to *E*. *faecalis* in *C*. *elegans* can also result from activating TRPM channels that mediate learned pathogen avoidance that causes intestinal distention (48).

Notably, hepatic HNF4α has now been implicated in polymicrobial sepsis-associated metabolic reprogramming, where it is required to prevent liver steatosis and organ damage while inducing liver regeneration, thereby decreasing the risk of death (49). Phenazines, a group of bacterial virulence factors, were identified as ligands for the aryl hydrocarbon receptor, which recognizes a wide array of endogenous ligands and environmental toxins and initiates immune responses in mammals (50). Intestinal tuft cells utilize taste and other metabolite receptors (including for succinate) that enables them to act as mucosal sentinel cells and activate type 2 immune responses (51). Other GPCRs have also been implicated in sensing self-metabolites (reviewed in ref. 2), in some cases participating in inflammasome activation, e.g., OLFR2 (52), in a manner that is compatible with the concept of coincidence detection. Interestingly, the NLRP3 inflammasome can respond to the microbial danger signals butyrate and propionate (53). Microbiota-derived metabolites, like butyrate, can modulate intestinal (type 2) immunity, for example, by restricting tuft cell differentiation (54).

Immune responses can be initiated locally by cell-autonomous perturbation of core cellular functions or in distant tissues from the initial site of homeostasis disruption, implicating non-cell-autonomous stress responses and interorgan communication in the mechanisms of surveillance immunity and promoting survival to environmental challenges that threaten the integrity of their genome, proteome, or metabolome (55). The stressed tissue may secrete factors that transmit signals to tissues in different organs and initiate processes that help the organism cope with stress. For example, evidence in *C*. *elegans* shows that the mitochondrial unfolded protein response (UPR^mt^) can be non-cell-autonomously mediated by Wnt signaling, which relays mitochondrial stress signals (“mitokines”) from neurons to peripheral tissues (56). Mild muscle mitochondrial distress in *D*. *melanogaster* initiates both local (redox-dependent induction of genes that regulate the UPR^mt^) and systemic responses (involving the transcriptional induction of the *Drosophila* ortholog of insulin-like growth factor–binding protein 7) that antagonize insulin signaling and promote mitophagy (57). IL-6 is induced in response to several forms of physiological disruption, including those caused by infection to coordinate systemic immunometabolic reprogramming; it behaves as a systemic stress hormone that mediates interorgan axis, such as those between brain/brown fat/liver (58). These and other types of responses cooperate to induce immune responses, cytoprotective pathways, and repair responses critical to dealing with stressors, ranging from environmental toxins to infectious challenges (59), that limit tissue damage and ultimately prolong lifespan.

We next explore examples of surveillance immunity in response to diverse types of homeostasis disruption at multiple organismal levels.

## Disruption of systemic and metabolic homeostasis

Arterial partial pressures of O_2_ and CO_2_, concentrations of K^+^, Ca^2+^, H^+^ (pH) and blood glucose, core body temperature, mean arterial pressure, blood volume, and blood osmolality are critical homeostatic variables. The organism monitors these variables and counters deviation using negative feedback mechanisms (33). Substantial deviations of these parameters have been documented to lead to inflammatory responses, as negative feedback mechanisms are insufficient to bring them back to their original physiological ranges.

### Low O_2_ (hypoxia) and glucose concentrations.

Low O_2_ (hypoxia) and glucose concentrations (which occur in pathological niches like tumors and infected or ischemic tissues, ref. 60) decrease the function of the rate-limiting enzyme of the mevalonate kinase pathway (HMG-CoA reductase, HMGCR), which leads to the activation of NLRP3 inflammasome (61). Stroke induces sustained inflammation and drives atherosclerosis by activating Notch1 in endothelial cells (62). Prolonged hypoxia can be sensed by pyridoxine 5′-phosphate oxidase (PNPO), an enzyme that catalyzes the bioactivation of vitamin B6, which decreases its activity under prolonged hypoxia, leading to deficient lysosome acidification and delayed resolution of the inflammatory response (63). Hypoxia is also a known cause of pulmonary hypertension because macrophages activate vascular remodeling (64).

### Hemodynamic perturbations.

Hemodynamic perturbations, like blood pressure increase, can cause microglial inflammatory activation, which can then act to control blood pressure changes (65, 66). The mechanically activated ion channels PIEZO1 and PIEZO2 are the critical sensors in baroreceptor neurons that monitor blood pressure to keep it in the appropriate physiological range (67). PIEZO1 is present and has demonstrated roles in macrophage-initiated inflammatory responses (68, 69). Notably, *Piezo1* deletion in the myeloid compartment decreases macrophage kidney infiltration and activation to prevent renal fibrosis, a common consequence of chronic hypertension (70).

### Thermoregulation.

Pathogens often disrupt thermoregulation by inducing either fever or hypothermia. Fever-range heat constitutes a danger signal that causes mitochondrial stress, resulting in the increase of T cell proliferation, migration, and inflammatory functions (35).

### Metabolic reprogramming.

Metabolic reprogramming in the presence of a pathogen may provide key signals to initiate and regulate immune responses (2). Sensing of substantial deviations in controlled metabolic fluxes may signal the presence of specific pathogen groups, because each has requirements that vary according to the specificities of their life cycles. Notably, blood glucose concentrations are modulated by infection. The host can sense these changes and potentiate innate antiviral immune responses (71) as well as metabolic defense strategies (72). A shift toward glycolytic-based metabolism is a hallmark of resistance mechanisms, while fatty acid oxidation and oxidative phosphorylation are central for disease tolerance mechanisms (2). Interestingly, acute suppression of mitochondrial ATP production prevents apoptosis and provides an essential signal of NLRP3 inflammasome activation (73), which may constitute an example of coincidence detection.

### Amino acid.

Amino acid availability can be sensed and interpreted by the host as the presence of a pathogen and constitutes a central regulatory node for immune responses and infection pathophysiology to bacteria and viruses. Host sensing of amino acid depletion induced by invasive bacterial pathogens initiates protective innate immune and stress responses, including by a decrease in mTOR activity leading to autophagy (74). Virus-dependent activation of GCN2, a conserved serine/threonine kinase that works as a stress sensor in response to amino acid deficiency, initiates autophagy and enhances antigen presentation to CD4^+^ and CD8^+^ T cells (75). Serine metabolism is critical in antiviral immunity and constitutes an integration hub for cellular metabolism, antiviral immunity, and epigenetic regulation. Deficiency of the amino acid serine promotes virus-induced IFN-β production (76). By contrast, increases in serine suppress interferon responses (77). Similarly, methionine restriction has been observed to limit tumor growth and to increase the responses to anticancer therapies (78).

### Nucleotide depletion.

Nucleotide depletion promotes cell fate transitions and induces DNA replication stress (79). In *C*. *elegans*, perturbations in purine metabolism are sensed and act as signals to promote defense against epithelial infection (80). Similarly, cellular pyrimidine deficiency triggers mitochondrial DNA–dependent innate immunity (81). NAD+ depletion is sensed by the innate immune sensor NLRC5 to trigger PANoptosis (a caspase and RIPK-driven inflammatory cell death mediated by PANoptosomes) and inflammation (82).

Both a decrease in cholesterol synthesis (83) and excess cholesterol concentrations (84, 85) have been causally linked to enhanced immune responses, suggesting that substantial deviations in cholesterol concentrations trigger inflammatory responses, possibly reflecting the targeting of cholesterol synthesis pathways by pathogens, especially viruses. Several mechanisms are likely to prevent deviations in cholesterol concentrations. Genetic (86) or pharmacologic (83) cholesterol synthesis inhibition by statins greatly increases interferon responses. By contrast, cholesterol excess directly causes mitochondrial DNA release and consequent activation of the AIM2 inflammasome, which can be prevented by producing 25-hydroxycholesterol in activated macrophages (84). Excess cholesterol has also been shown to promote adipose tissue inflammation (87). Oxidized lipids (OLs) may serve as generic indicators of threat to the host, both in the context of sterile and septic injury. OLs resulting from tissue injury caused by infection can act as immunomodulatory signals, leading to pro- or antiinflammatory downstream responses, depending on the context (88). Similarly, in *Drosophila*, sugar alcohols of the polyol pathway may serve as alarmins and mediate communication between local and systemic innate immune responses (89).

## Disruption of cellular, organellar and molecular homeostasis

We have previously documented evidence supporting a critical role for the disruption of homeostasis at the cellular, organellar, and molecular levels in shaping the initiation and quality of immune responses (reviewed in ref. 3). In Table 1, we summarize, update, and document the clinical implications of these observations. Key cellular processes, such as energy metabolism and protein production, are compartmentalized within organelles. Damage to organelles can cause leakage of contents that activate sensors that detect misplaced molecules. Such disruptions can result from infections or sterile conditions, like autoinflammatory diseases. Stresses like infections, mechanical strain, or nutrient changes disrupt organelle integrity, triggering repair programs to restore balance. Persistent damage often leads to low-grade inflammation (parainflammation), while transient stress mechanisms that promote inflammation remain less understood (34). Emerging evidence highlights mitochondria, ER, lysosome, Golgi, and nuclear envelope stress as sources of proinflammatory signals (Table 1). Targeting pathways that restore cellular, organellar, and molecular homeostasis or mitigate stress responses offers therapeutic potential for infections and chronic inflammatory diseases.

## Conclusions and future perspectives

Initiation of immune responses following the distinction between pathogens and commensal microorganisms is likely to require direct sensing of microorganisms in the context of the physiological perturbations they cause. Integration of this information may be accomplished at the molecular level by coincidence detectors that signal for downstream events only when both types of signals are present. There are many open questions posed by a model of surveillance immunity (Table 2), including (a) the identity of upstream sensors of pathogen-disrupted physiological parameters; (b) how these signals molecularly integrate with the information coming from direct microorganism sensing; (c) the identity of coincidence detectors; and (d) the downstream signaling events leading to resistance and disease tolerance processes. This knowledge is not only of biological interest but has important clinical implications, including the distinction between colonization and infection. It can also potentially inspire novel therapeutic strategies to treat severe infection and chronic inflammation.

Inflammation is a core component of most, if not all, known pathologic chronic conditions (39). It has historically been considered a response to septic and aseptic injury, but it may have evolved as an adaptive response for restoring homeostasis, as it is now clear that inflammation is likely to have additional critical physiological roles, including in the orchestration of the feed-forward mechanisms that deal with severe disruption in homeostasis that cannot be restored by negative feedback mechanisms (34). Macrophages are likely to have central roles in sensing physiological disruption that initiates inflammatory responses (90).

If, as we argue here, sensing of homeostasis disruption caused by the presence of a pathogen is critical, perhaps sufficient, to initiate immune responses, it is conceivable that the persistence of physiologically monitored parameters outside of their normal ranges may cause systemic chronic inflammation that favors the occurrence of the highly prevalent modern chronic conditions, including type 2 diabetes and cardiovascular diseases. In this context, for example, maintaining high blood pressure or nontreated sleep apnea, which causes repeated decreases in O_2_ saturation, would promote the initiation and progression of cardiovascular disease mainly because they cause persistent systemic inflammation. Other forms of persistent nonresolved nutritional and metabolic stress (91) could lead to many of the other current major health concerns, including obesity and metabolic dysfunction–associated steatohepatitis (92). In addition, chronic inflammation can be a critical contributing factor to many cancer types. Notably, inflammation is not routinely treated in these conditions, but several observations, including in human clinical trials, suggest that targeting inflammation may be highly effective in preventing acute cardiovascular events, including myocardial infarction (93) and stroke (94).

An additional important clinical implication of a model of surveillance immunity is the need for research to understand the effects of many drugs used routinely by hundreds of millions of people, either to explore their nonconventional effects or to minimize their undesirable side effects. For example, clinicians have known and empirically preferentially used classes of antibiotics that better resolve an infection than would be expected from their direct antimicrobial activity alone and are superior to different classes with overlapping antimicrobial spectra. In addition to their direct antibacterial activities, these antibiotics have been demonstrated to have off-target host effects that cause physiologic perturbations, including mitochondrial protein synthesis inhibition and DNA damage that trigger the production of immune mediators (often interferon-stimulated genes) or limit tissue damage caused by the infection or associated immune response, thereby promoting organ function (reviewed in ref. 2). Notably, antibiotics, like tetracyclines, are among the most prescribed drugs for dermatological conditions and act via mechanisms independent of their antibiotic activity, which likely rely on their ability to cause physiologic perturbations. Statins, which block cholesterol synthesis, modulate immune responses and, therefore, may impact cardiovascular diseases beyond their well-known direct cholesterol-lowering properties. Another example is that of highly prescribed drugs that affect the function of the mitochondrial ETC, like metformin. ETC perturbations have been shown to modify the progression of a severe infection (95). These properties should be further explored in relation to effects on infection susceptibility and vaccine effectiveness. We can also expect that cancer chemotherapeutic drugs that affect nucleic acid homeostasis, particularly DNA and chromatin, and cell death pathways with DAMP release, dramatically affect immune (96) and inflammatory responses (97), which may be explored to get more effective antitumoral responses and decrease resistance to standard therapy (98), in addition to the management of undesired side effects (99).

Models of immune response initiation, particularly those relying solely on the direct detection of microorganisms, inadequately describe the pathophysiological mechanisms governing the initiation, progression, and resolution of immune responses. Models that consider homeostasis disruption as a key factor in immune response initiation are necessary not only because they add considerably to our understanding of organismal physiology, but also because they have broad implications for human pathophysiology and effective treatment of acute infection and chronic disease.

## Figures and Tables

**Table 2:**
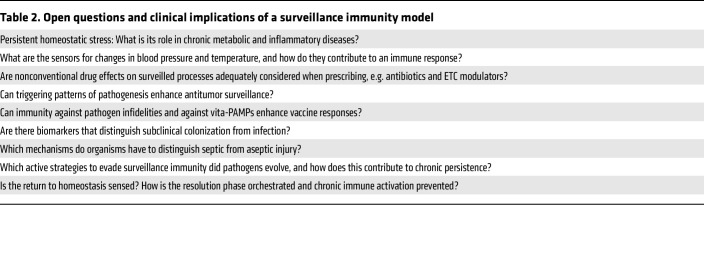
Open questions and clinical implications of a surveillance immunity model

**Table 1:**
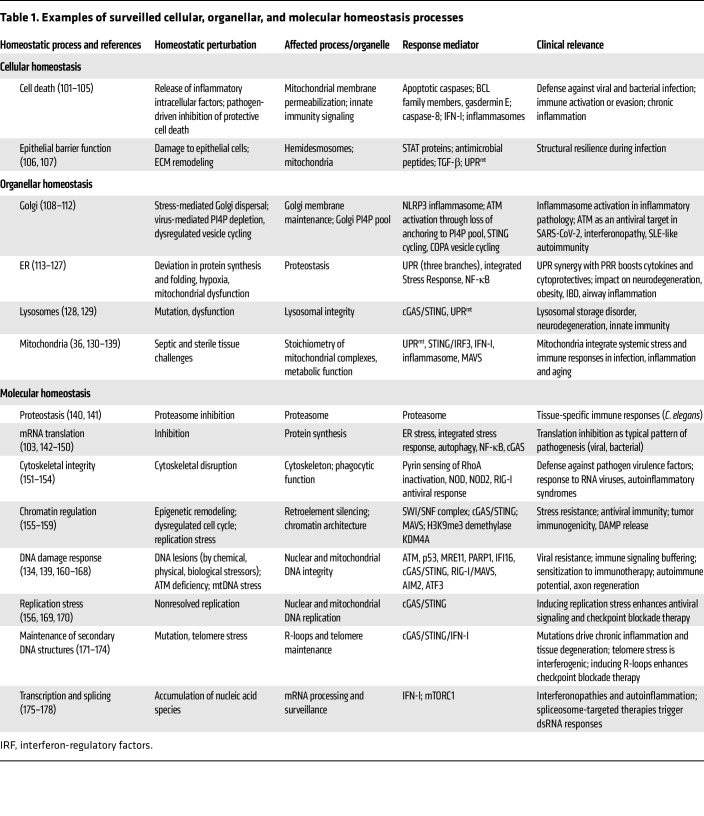
Examples of surveilled cellular, organellar, and molecular homeostasis processes

## Version 1 08/01/2025

Electronic publication

## Version 2 08/04/2025

Corrected author name in ref. 6
